# A Case of COVID-19 Related Coagulopathy Complications and Heparin Resistance

**DOI:** 10.7759/cureus.18265

**Published:** 2021-09-25

**Authors:** Erum Chowdhry, Jennifer Moshman, Stacey Carroll

**Affiliations:** 1 Family Medicine, University of Nevada, Reno School of Medicine, Reno, USA; 2 Internal Medicine, Providence Newberg Medical Center, Newberg, USA

**Keywords:** anticoagulation, covid coagulopathy, coagulopathy, heparin resistance, covid-19

## Abstract

Mechanisms of COVID-19 coagulopathy have been speculated and are not definitively understood; the current speculation is that there is elaborate crosstalk between the inflammatory and hemostatic systems which contributes to the overall increased thrombotic risk in the setting of COVID-19 resulting in a hypercoagulable state. A few documented reports regarding cases of apparent heparin resistance in patients with COVID-19 with complications of thromboembolic events occurring in the setting of heparin anticoagulation have been described. This phenomenon of heparin resistance has been observed in patients with active, severe COVID-19 infection. However, we describe a unique case of a patient who had recovered from a recent, mild COVID-19 infection that did not require hospitalization and presented with acute limb ischemia and demonstrated heparin resistance. The patient was managed by specialists in vascular surgery, intensivists, cardiologists, hematology, and physical medicine and rehabilitation (PMR). We present the case of a patient who had successfully recovered from COVID-19 yet demonstrated post-COVID-19 complications related to coagulopathy and heparin resistance.

## Introduction

COVID-19 causes an exaggerated inflammatory response which leads to severe complications such as acute respiratory distress syndrome, acute respiratory failure, sepsis, pneumonia, coagulopathy, and death. Among those with severe COVID-19 complications, coagulopathy has been reported in up to 50% of patients [1]. Evidence suggests that an increase in D-dimer levels proportionately correlates to a worse prognosis [1,2]. Certainly, the coexistence of other comorbidities such as obesity and cardiovascular disease, as well as elevated C-reactive protein, troponins, and other disseminated intravascular coagulation markers are also associated with a worse prognosis in hospitalized COVID-19 patients [2].

It has been nearly two years since the first outbreak of COVID-19 started in Wuhan, Hubei, China in November 2019, and the pandemic continues. The unique presentations of COVID-19 infection have been documented with varying severity and symptom occurrences. With an increasing number of patients having recovered from COVID-19, we have also come to know of the post-viral syndrome in which patients experience long-term health consequences and symptoms even after testing negative for the infection. Of the potential complications, coagulopathy has been well described in cases of active COVID-19 infection [3,4]. However, in this case report, we describe the complications of coagulopathy in a patient who had recently recovered from a mild COVID-19 infection that did not require hospitalization. 

## Case presentation

An obese 33-year-old male patient with no significant past medical history presented to the emergency room (ER) complaining of left-leg pain after a recent COVID-19 infection. He had tested positive nearly three weeks earlier and had remained asymptomatic, not requiring hospitalization. Repeat testing on admission via antigen and polymerase chain reaction (PCR) was negative. He developed acute onset severe pain and swelling in the left leg and foot nearly one week before presentation, which progressed to numbness. He did not seek medical attention previously until the current presentation when his pain became unbearable. Five days before arriving at the ER, he also had a motor loss of the toes and ankle. The patient denied any coughing, had no shortness of breath or chest pain. The patient was afebrile and vital signs were stable on presentation. On physical exam, the patient had positive Homan’s sign and palpable cord of the left lower extremity with minimal swelling. The right and left dorsalis pedis (DP) and posterior tibial (PT) pulses were palpable. The popliteal pulses were palpable on the right side and noted to be monophasic on the left. The femoral pulses were palpable bilaterally. The left foot was noted to be cool in temperature with diminished sensation at the level of the ankle. The patient also had a foot drop, was unable to flex the ankle, minimal toe flexion/extension, and early mottling of the skin was noted. The rest of the physical exam was within normal limits. 

**Figure 1:**
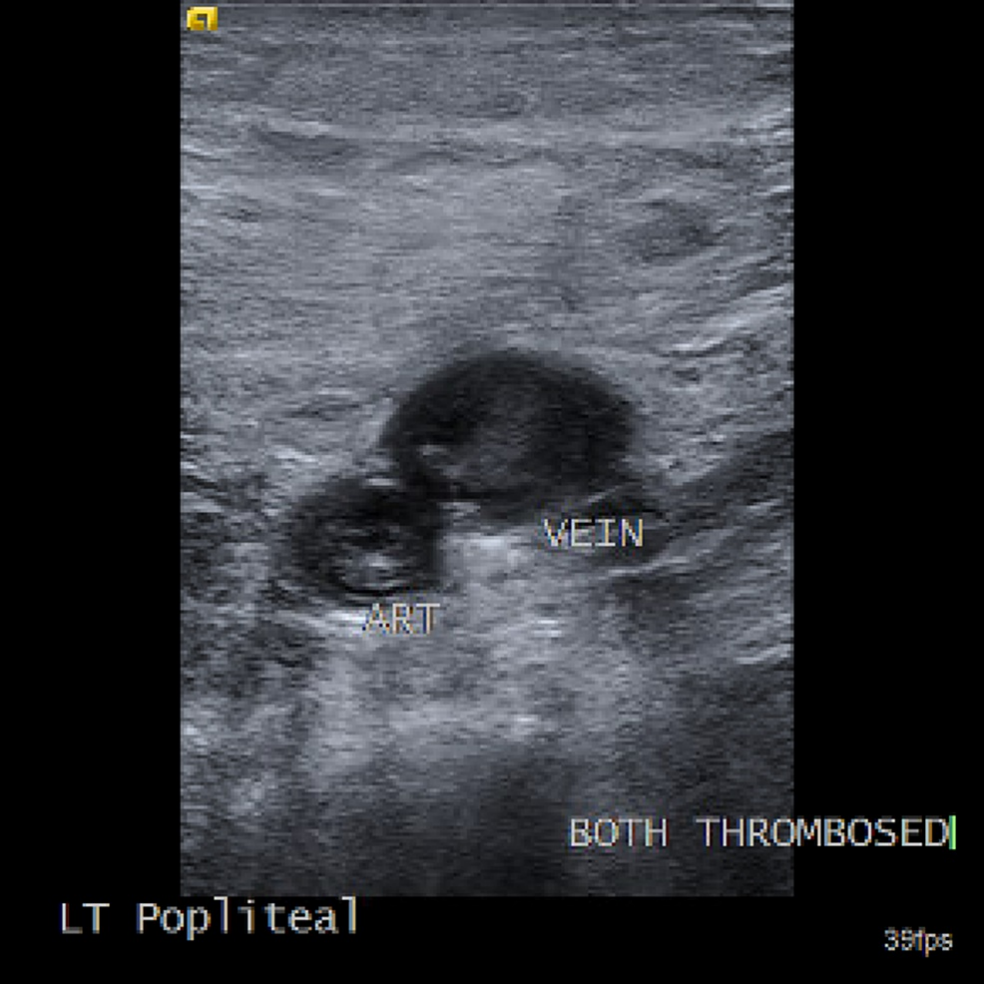
Left popliteal artery and vein, visualized in the axial view of ultrasound, are both thrombosed.

Ultrasound of the left lower extremity showed evidence of acute deep venous thrombosis in the popliteal (partial) and gastrocnemius (nearly occlusive) veins. Subcutaneous edema and rouleaux flow were seen throughout the extremity. Nearly occlusive arterial thromboses were also discovered throughout the distal femoral, popliteal, posterior tibial, anterior tibial, and peroneal arteries with very low flow velocities to absent flow overall (Figure 1). More proximally, triphasic waveforms were observed with moderately reduced velocities through the common femoral, deep femoral, proximal, and mid-femoral arteries. Heparin infusion was immediately started. Vascular surgery was consulted, and the patient was taken to the operating room for an open thrombectomy of the superficial femoral artery, popliteal, anterior tibial, posterior tibial, and peroneal arteries under general anesthesia. Heparin infusion was maintained throughout the procedure and the patient was also heparinized using 100 U/kg heparin which circulated for three minutes before the activated clotting time (ACT) was measured. The ACT was maintained between 250-300 throughout the procedure. Despite appropriate anticoagulation, he had recurrent thromboses. The posterior tibial artery lost signal within a few minutes of closing and was reoccluded. These tibial vessels were subsequently reopened, and he underwent repeat thrombectomy. After the re-thrombectomy, the patient developed signs and symptoms of impending respiratory failure with oxygen saturations dropping down to the low 70s despite a 100% fraction of inspired oxygen (FiO2) and tachycardia. As such, the patient was intubated. The posterior tibial artery was reoccluded, but there was a signal in the dorsalis pedis. However, because the patient was deemed to be in danger of significant decompensation, a third thrombectomy was not attempted.

**Figure 2:**
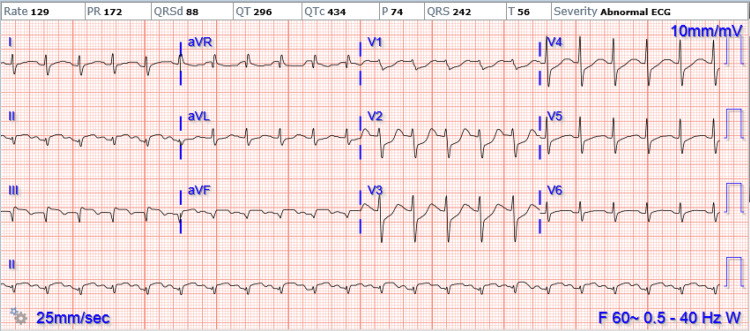
EKG showing evidence of acute infarct in inferoposterior distribution.

Post-operatively, the patient developed worsening respiratory failure, increased work of breathing, and apparent ST elevation on the monitor. EKG confirmed an inferior ST segment elevation myocardial infarction (STEMI) with reciprocal anterior ST depression (Figure 2). Unfortunately, the doppler signal of the anterior tibial artery was also lost around the same time. He was taken emergently to the cardiac catheterization lab and was deemed unstable for return to the OR for another attempt at tibial thrombectomy. 

In the cardiac catheterization lab, he was found to have triple vessel disease; percutaneous coronary intervention was attempted but was unsuccessful. The ejection fraction based on the left ventriculogram was 20%. He experienced atrial flutter during the procedure requiring chemical cardioversion with amiodarone. He was noted to have a possible left apical and right mural thrombus both of which were confirmed on a subsequent echocardiogram. His left foot remained cold and pulseless. Patient remained on a heparin gutta (gtt) (heparin infusion 25,000 units in 500 mL 0.45% NaCl continuous at 0-30 units/kg/hr, titrated per protocol while monitoring activated partial thromboplastin time [aPTT]). Clopidogrel (Plavix) 300 mg per os (PO) was administered once after the patient sustained a STEMI and then continued at 75 mg PO daily thereafter. The patient demonstrated signs of heparin resistance as he could not reach goal aPTT despite maximal heparin gtt with a mean activated partial thromboplastin time of 55.7 seconds and an average anti-Xa assay of 0.24 IU/mL. Argatroban infusion was initiated at 2 mcg/kg/min and aPTT level within 24 hours of initiating argatroban was 114.8 seconds, achieving the therapeutic range indicated for anticoagulation. His left foot was not deemed salvageable and given his poor overall condition, ongoing hypercoagulability, and mortality risk, a guillotine amputation of the left foot just proximal to the ankle was performed. No arterial inflow was present. An acute clot was extracted from the posterior tibial artery with the restoration of pulsatile inflow. The clot was also extracted from the anterior tibial artery with the return of weak inflow. 

Four days following the STEMI, the patient developed pulseless ventricular tachycardia and was successfully resuscitated and started on amiodarone. While he remained on mechanical ventilation, he required intermittent pressor therapy for undifferentiated shock. Three days later, the patient was successfully extubated and transferred out of the ICU and began to work with physical therapy. One week later, the patient developed recurrent acute hypoxemic respiratory failure, requiring re-intubation, and he was transferred back to the ICU. Anticoagulation with warfarin was initiated after bridging with fondaparinux. Three days later, the patient was extubated and then three days thereafter, he again went into acute respiratory distress, requiring BiPAP. Despite attempts at non-invasive ventilation using BiPAP, he became somnolent and required intubation, and transition to full mechanical ventilatory support was altered and he was intubated. During the peri-intubation period, the patient lost pulse and went into pulseless electrical activity and advanced cardiac life support (ACLS) protocol was initiated. Thirty minutes of ACLS was completed and a cardiac ultrasound thereafter showed no cardiac movement with the presence of a ventricular thrombus. The patient was pronounced dead. 

Hematology-oncology was consulted during the hospitalization to investigate potential underlying etiologies of the patient’s hypercoagulability, given his young age and unusual clotting symptoms. Further lab workup to investigate causes of hypercoagulopathy included antiphospholipid antibodies, anticardiolipin antibodies, beta 2 microglobulin antibody, lupus anticoagulant, and factor II DNA analysis that all resulted negative. Flow cytometry testing for paroxysmal nocturnal hemoglobinuria was also collected and the results were negative. Janus Kinase 2 (JAK2) kinase mutation looking for evidence of polycythemia vera or other myeloproliferative disorders was also negative. Other hypercoagulable syndromes more related to venous clotting, specifically with factor V Leiden, prothrombin gene mutation, protein C deficiency, protein S deficiency, and antithrombin III deficiency were worked up (although it was expected that the antithrombin III level was not reliable, as the patient remained on heparin in the setting of acute clotting) and no test came back positive. Screening for underlying connective tissue disorders/rheumatologic disorders was conducted inclusive of the antinuclear antibody (ANA), rheumatoid factor, and serum protein electrophoresis (SPEP) with quantitative immunoglobulins. This extensive workup for hypercoagulability, while the patient was hospitalized, was non-diagnostic, suggesting COVID-19 syndrome of hypercoagulability as the most probable etiology in the post-acute infection setting. 

The patient also notably had elevated blood glucose levels during his hospitalization ranging in the 200s. An A1C was then obtained and resulted to be 12.0%. Though the patient had not been formally diagnosed with diabetes, his hemoglobin A1c (HbA1C) and elevated blood glucose readings in the hospital confirmed the diagnosis of type 2 diabetes. His blood glucose levels were controlled in the inpatient setting with sliding scale insulin. 

## Discussion

Interestingly, this patient had recovered from his COVID-19 infection and presented with complications of coagulopathy in the setting of post-infection. Events of COVID-19-associated coagulopathy have been reported in patients with active, severe infection; however, post-COVID-19 coagulopathy has not been as commonly described.

Most patients who are critically ill with COVID-19 in the hospital are anticoagulated with low molecular weight heparin or unfractionated heparin, including those patients who develop thrombotic events. Heparin resistance has been documented in severely ill, COVID-19 patients in the ICU [5-7]. However, the phenomenon has not been commonly reported in patients who have recovered from infection. In the setting of arterial and venous thromboses as was seen in this patient, it is critical to achieve therapeutic heparinization for limb salvage and to prevent mortality. However, when target aPTT or anti-factor Xa levels are not achieved when on therapeutic doses of unfractionated heparin, this phenomenon is called heparin resistance and has been described in hospitalized patients with COVID-19. Although the prevalence of anticoagulation failure in COVID-19 patients is not clear, a small retrospective study conducted by Llitjos et al. found that more than 50% of patients receiving therapeutic anticoagulation on heparin underwent progressive thrombosis [8]. 

The mechanisms behind heparin resistance in COVID-19 patients are not understood and require further clinical and laboratory studies. Certainly, the presence of comorbidities and/or risk factors such as obesity and diabetes which were both present in the patient have implications in hypercoagulability. In obese individuals, lifestyle factors such as decreased mobility contribute to an increased risk of deep vein thrombosis (DVT). Additionally, obesity also promotes chronic inflammation and impaired fibrinolysis, both of which contribute to an increased risk of thrombosis [8]. The vascular complications of diabetes are well known, and the underlying chronic hyperglycemic state certainly contributes to inflammation, endothelial dysfunction, and hypercoagulability [9]. Type 2 diabetes is also associated with various coagulopathies with evidence that there is an upregulation of specific cytokines and circulating inflammatory markers which are contributory factors to hypercoagulation and abnormal platelet activation [9-11]. One retrospective study by Wang et al. specifically looked at the glycosylated hemoglobin levels and found a statistically significant positive correlation with high HbA1c levels and inflammation, hypercoagulability, and mortality rate in diabetic patients with COVID-19 [12]. 

Furthermore, another study by White et al. found that heparin resistance in COVID-19 patients did not correlate with increased fibrinogen or factor VIII or with decreased antithrombin. Rather, considering molecularly, heparin is negatively charged and thus, has the potential to interact with positively charged plasma proteins, which behave similarly to acute phase reactants. Therefore, these acute phase reactants are essentially competing for heparin-binding [13]. In inflammatory states such as in the setting of COVID-19 infection, acute phase reactants are increased and thus biomechanically, it could account for competitive inhibition and hence account for the observed heparin resistance. However, this proposed mechanism is yet to be confirmed [13]. 

Alternative anticoagulative agents to heparin have been used consequently in the setting of heparin resistance. In this patient, anticoagulation was switched to argatroban, a direct oral anticoagulant (DOAC), and there is evidence that adequate anticoagulation has been achieved with argatroban [5,14]. It has also been speculated that DOAC therapy may yield a theoretical advantage since its mechanism is free from antithrombin [15]. Limited studies have looked at the efficacy of other anticoagulative agents used in hospitalized COVID-19 patients and concluded that apixaban has similar efficacy to enoxaparin in decreasing mortality in those with moderate or severe illness from COVID-19 [16].

## Conclusions

Further studies are required to investigate the management of thrombotic events in patients with COVID-19 as well as for thromboprophylaxis after acute COVID-19 infection and when outpatient prophylaxis is indicated, taking into account the individualized risks of thrombosis against the risk of bleeding. Additionally, research into the efficacies of various anticoagulants needs to be further investigated, especially in the setting of increased reports of heparin resistance in hospitalized patients. With the increased number of recovered COVID-19 patients, we anticipate more data on studies looking into antithrombotic therapies for patients with acute infection and post-infection. Therapeutic guidelines are being proposed and revised as more data becomes available and further studies of the mechanisms will contribute to our evolving understanding of COVID-19 related complications.
